# Cortex Phellodendri Extract Supplementation Alleviates Intestinal Oxidative Stress, Inflammation, and Microbiota Dysbiosis in Early Weaning Piglets

**DOI:** 10.3390/ani16162628

**Published:** 2026-08-21

**Authors:** Xinran Huang, Ting Yang, Wenxia Qin, Libao Ma, Caimei Yang, Baoyang Xu

**Affiliations:** 1 College of Animal Science and Technology, College of Veterinary Medicine, Zhejiang A&F University, Hangzhou 311300, China; 2 College of Animal Science and Technology, Huazhong Agricultural University, Wuhan 430070, China

**Keywords:** Cortex Phellodendri extract, oxidative stress, inflammation, microbiota dysbiosis, early weaning piglets

## Abstract

Piglets’ early weaning frequently encounters intestinal dysfunction and diarrhea that results in growth retardation and even mortality. We previously found Cortex Phellodendri extract (CPE) could relieve post-weaning piglets’ diarrhea whereas the underlying mechanism remains unclear. Here, to explore the mechanism, 126 piglets were allotted into three groups of seven replicates fed with basal diet, or basal diet + 0.04% CPE, or basal diet + 0.08% CPE. We found 0.04% CPE supplementation effectively decreased piglets’ fecal scores on d7 and d14 post-weaning. In addition, CPE supplementation improved growth performance of early weaning piglets during days 8–14. Further analysis showed CPE supplementation improved the jejunum morphology and increased antioxidant indices, whereas it reduced oxidative metabolites in jejunum tissue. Meanwhile, CPE supplementation increased the mRNA levels of antioxidant indices and anti-inflammatory cytokines but decreased the mRNA levels of inflammatory cytokines. CPE supplementation shifted gut microbiota and increased alpha diversity indices, namely the Observed species and Abundance-Based Coverage Estimator indices. CPE supplementation increased the relative abundances of Proteobacteria, *SMB53*, *Flexispira*, and *Ruminococcus_gnavus*, but decreased the relative abundances of Parabacteroides and *Sutterella*. The gut microbiota shifted by CPE supplementation has a significant correlation with the early weaning-related indices. Overall, CPE supplementation alleviated intestinal oxidative stress, inflammation, and microbiota dysbiosis in early weaning piglets.

## Simple Summary

Early weaning in piglets frequently leads to intestinal dysfunction and diarrhea, resulting in growth retardation and even mortality. In this study, we investigated the potential of Cortex Phellodendri extract (CPE) to alleviate intestinal oxidative stress, inflammatory responses, and gut microbiota dysbiosis in early weaning piglets. Our results demonstrated that dietary supplementation with 0.04% CPE significantly reduced fecal scores on days 7 and 14 post-weaning, while also improving growth performance during days 8–14 post-weaning. Moreover, CPE supplementation enhanced jejunal morphology, elevated intestinal antioxidant enzyme activities, and concurrently decreased oxidative metabolite levels. At the immune level, CPE administration upregulated anti-inflammatory cytokine expression and downregulated pro-inflammatory cytokine expression. Notably, CPE treatment reshaped the gut microbial community and increased its alpha diversity. Furthermore, the CPE-induced microbial shifts exhibited significant correlations with key early weaning parameters. Collectively, these findings indicate that CPE supplementation effectively attenuates oxidative stress, inflammation, and microbial dysbiosis in the intestines of early-weaned piglets.

## 1. Introduction

The weaning age of piglets is practiced at three to four weeks in modern swine production system although the natural weaning age is about 17 weeks [1]. Weaning is a critical period in pig production, during which piglets are exposed to a combination of psychological, environmental, and physiological stressors [2]. The early post-weaning phase is especially challenging, often marked by a temporary reduction in feed intake, disruption of the intestinal microbial community, impairment of gut barrier integrity, and increased susceptibility to enteric infections and diarrheal diseases—all of which collectively impair subsequent growth performance [3,4]. To mitigate these weaning-associated challenges, conventional practices have heavily relied on supplementing diets with pharmacological levels of zinc and copper, along with widespread use of antimicrobial agents. However, these strategies have drawn growing concern due to their contribution to environmental pollution and the accelerating threat of antibiotic resistance [5]. With the expansion of the ban on the use of antibiotic growth promoters for disease prevention and growth promotion in swine production, as well as restrictions on copper and zinc additions, searching for antibiotic alternatives becomes urgent [2,6,7,8,9].

To ameliorate piglets’ early weaning stress, traditional Chinese herbal medicines have attracted growing interest as promising candidates to replace conventional antibiotics for alleviating early weaning stress in piglets, owing to their low toxicity and comparable or superior efficacy in supporting intestinal health [10,11]. Among these, Cortex Phellodendri—known as “Huang Bai” in Chinese—yields an extract (CPE) that is rich in berberine, which serves as its principal bioactive component [12,13]. For centuries, this herbal remedy has been widely used in Chinese clinical practice to manage gastrointestinal infections caused by pathogenic bacteria, particularly bacterial diarrhea and dysentery [12,13]. However, the underlying mechanism by which CPE exhibits pharmaceutical effect remains poorly understood. That is mainly because CPE has broad-spectrum but has weak antimicrobial activity [12]. In addition, little CPE can enter the bloodstream from the intestine because CPE is mainly absorbed through the intestine whereas the absorption rate and bioavailability are both very low [14]. A growing body of evidence highlights the involvement of gut microbiota in mediating the therapeutic effects of traditional Chinese medicines [10,15,16]. In mammals, the intestinal microbial community is closely intertwined with host physiology, influencing immune regulation, defending against pathogens [17,18,19], and playing a role in the progression of colitis and other infectious conditions [20]. Our previous work further demonstrated that CPE administration could modulate gut microbial composition in a dose-dependent manner, thereby protecting mice from diarrhea induced by enterotoxigenic *Escherichia coli* [21].

Moreover, we previously found that CPE can effectively ameliorate post-weaning piglets’ diarrhea [22]. However, the underlying mechanism by which CPE exhibits anti-diarrhea activity and its effect on ameliorating piglets’ early weaning stress remain unclear. Here, we therefore evaluate the effect of dietary CPE supplementation on alleviating intestinal oxidative stress, inflammation and microbiota dysbiosis in early weaning piglets.

## 2. Materials and Methods

### 2.1. Ethical Statement

All procedures involving animals were reviewed and approved by the Animal Experimental Ethical Inspection Committee of the Laboratory Animal Centre at Huazhong Agricultural University (approval number HZAUSW20210012, the ethics approval date 22 October 2021).

### 2.2. Materials and Chemicals

The CPE used in this study was obtained from Hengrui Tongda Bio. Co. Ltd. (Chengdu, Sichuan, China). Its preparation followed the protocols outlined in the 2015 edition of the Veterinary Pharmacopeia of the People’s Republic of China. The berberine content in the CPE was quantified at 21.75% (*w*/*w*) using high-performance liquid chromatography (HPLC) on a U-3000 (Thermo Scientific, Waltham, MA, USA) system equipped with a reversed-phase Hypersil GOLD™ LC column (250 mm × 4.6 mm, 5 μm particle size, Thermo, USA). Quantification was performed by comparing peak areas against a reference standard of berberine hydrochloride provided by the China Institute of Food and Drug Determination, employing the external standard method [21].

### 2.3. Animals and Experimental Treatments

A total of 126 early-weaned piglets (Landrace × Yorkshire, 23 ± 1 days of age, initial body weight 6.32 ± 0.24 kg) were randomly assigned to three dietary treatments, each consisting of seven replicate pens with six piglets per pen (the temperature 28–32 °C and the humidity 60~80%). Over a 14-day post-weaning period (days 0–14), the piglets received either a basal diet (Ctrl group), or the basal diet supplemented with 0.04% CPE (0.04% CPE group), or the basal diet supplemented with 0.08% CPE (0.08% CPE group). During the experiment, the early weaning piglets in each group were free to eat and drink. Piglets were vaccinated against porcine circovirus at around 7 days of age and against porcine mycoplasma pneumonia (respiratory disease) at 14 days of age. The basal diet was formulated to meet the nutritional requirements specified by the National Research Council (NRC, 2012), with its detailed composition provided in Table S1. Based on our prior study, which demonstrated that 0.03% CPE supplementation was able to alleviate post-weaning diarrhea [21], the 0.04% group was selected as the primary treatment group for mechanistic investigation; the 0.08% group was included as a high-dose safety check.

### 2.4. Piglet Growth Performance and Fecal Scores

To evaluate growth performance, we recorded the average daily gain (ADG) and average daily feed intake (ADFI) of each piglet throughout the trial. Fecal scores were assessed daily using a four-point scoring system, ranging from 1 (normal, well-formed feces) to 4 (severe, watery diarrhea), as previously described [23,24]. To reduce subjective error, the fecal score of piglets was calculated by averaging the scores from three independent observers.

### 2.5. Sample Collection

On day 14 post-weaning, based on the observed effects of 0.04% and 0.08% CPE supplementation on fecal scores, the control (Ctrl) and 0.04% CPE (CPE) groups were selected for the slaughter experiment. One piglet per replicate from each of these two groups was then randomly chosen for sample collection. Piglets were euthanized by intravenous injection of sodium pentobarbital (50 mg/kg body weight). Three 3 cm long segments were removed from middle locations of the small intestine, one was placed in 4% paraformaldehyde for histological evaluation, and the other two segments were stored at −80 °C after freezing in liquid nitrogen. After homogenization, colonic contents were collected and then stored at −80 °C after freezing in liquid nitrogen.

### 2.6. Histomorphology Examination of the Jejunum Tissue

To preserve the jejunum samples, tissues were fixed overnight in 4% paraformaldehyde at 4 °C. Following fixation, the specimens underwent graded ethanol dehydration (70–100%) and were subsequently embedded in paraffin wax. The resulting blocks were cut into 7 μm sections, which were mounted onto glass slides and incubated at 56 °C for 24 h to ensure firm adhesion. After dewaxing and rehydration, the slides were stained with hematoxylin and eosin. Morphological evaluation was carried out using an Olympus BX51 microscope equipped with a digital imaging system (Olympus Co., Tokyo, Japan). Villus height was measured as the straight-line span from the crypt-villus junction to the villus tip, while crypt depth was recorded as the distance from the crypt base to the junction at the crypt opening.

### 2.7. Antioxidant Indices Determination of the Jejunum Tissue

Commercial assay kits (Solarbio Science & Technology, Beijing, China) were used to measure the levels of several oxidative stress and antioxidant parameters in jejunal tissues, including total antioxidant capacity (T-AOC), the glutathione (GSH) to glutathione disulfide (GSSG) ratio, malondialdehyde (MDA), catalase (CAT), and nitric oxide (NO).

### 2.8. The mRNA Expression of Inflammatory Factors and Apoptosis Genes in Jejunum Tissue

Total RNA was isolated from jejunal tissues using TRIzol reagent (Invitrogen, Carlsbad, CA, USA), and its purity and concentration were assessed with a NanoDrop 2000 spectrophotometer (Thermo Scientific, Waltham, MA, USA). Subsequently, 2 µg of the extracted RNA served as the template for first-strand cDNA synthesis, which was carried out with a reverse transcription kit (Thermo Scientific, Waltham, MA, USA) following the supplier’s recommended protocol. Quantitative real-time PCR was then conducted on a CFX384 system (Bio-Rad, Hercules, CA, USA) using iTaq Universal SYBR Green Super Mix (Bio-Rad, Hercules, CA, USA). The thermal cycling conditions included an initial denaturation at 95 °C for 5 min, followed by 40 cycles of 95 °C for 15 s, 60 °C for 30 s, and 72 °C for 30 s, with a final elongation step at 72 °C. Relative transcript levels were calculated using the 2^−ΔΔCt^ method and normalized against the endogenous reference gene GAPDH. Relative transcript abundances were normalized to the internal reference gene GAPDH. All primer sequences are provided in Table S2.

### 2.9. Gut Microbial DNA Extraction and Sequencing

Microbial genomic DNA was extracted from colonic content samples using the Stool DNA Kit (TIANGEN, Beijing, China). The V4 hypervariable region of the bacterial 16S rRNA gene was amplified via PCR with the universal primer pair 515F (5′-GTGYCAGCMGCCGCGGTAA-3′) and 806R (5′-GGACTACNVGGGTWTCTAAT-3′). Following purification, the resulting amplicons were pooled in equal molar ratios and subjected to paired-end sequencing (2 × 250 bp) on a NovaSeq 6000 platform (Illumina, San Diego, CA, USA) at Biomarker Technologies Co., Ltd. (Beijing, China).

### 2.10. Gut Microbial Data Analysis

Raw sequencing reads were processed using the QIIME2 pipeline (v.2021.6) for initial quality control and feature table construction [25]. USEARCH (v.10.0) was then applied to cluster high-quality sequences into operational taxonomic units (OTUs) at a 97% similarity threshold [26]. Taxonomic assignment for each OTU representative sequence was performed using the RDP Classifier, with reference to the Silva (Release 132) and Greengenes (v.13.5) 16S rRNA databases [27,28]. Alpha diversity metrics, including Observed species, Chao1, Abundance-Based Coverage Estimator (ACE), Shannon, and Simpson indices, were computed using Mothur (v.1.31.2) [29]. Beta diversity was assessed by principal coordinate analysis (PCoA) based on Bray–Curtis dissimilarities, and statistical differences among groups were tested using permutational multivariate analysis of variance (PERMANOVA) with 999 Monte Carlo permutations, both implemented via the ‘vegan’ package in R (v.4.3.1) [30].

### 2.11. Data Processing and Statistics Analysis

Statistical analyses were performed using GraphPad 8.0 software. Comparisons between two groups were conducted using two-sided Student’s *t*-tests, while comparisons among multiple groups were analyzed using ANOVA followed by the Duncan multiple comparison test. Results were presented as mean ± SD and the significance was presented as * *p* < 0.05, ** *p* < 0.01, and *** *p* < 0.001.

## 3. Results

### 3.1. Dietary CPE Supplementation Attenuated Early Weaning Piglets’ Diarrhea

As shown in Table 1, compared with the Ctrl group, 0.04% CPE and 0.08% CPE decreased piglets’ fecal scores on d7 (*p* < 0.05) and d14 post-weaning (*p* < 0.05). There is no difference in attenuation of early weaning piglets’ diarrhea between 0.04% CPE and 0.08% CPE. Therefore, 0.04% CPE supplementation was an effective dose and was assigned to the CPE treatment group (CPE) for the subsequent analysis.

### 3.2. Dietary CPE Supplementation Improved the Growth Performance of Early Weaning Piglets

As shown in Table 2, CPE supplementation had no significant effect on early weaning piglets’ growth performance on d7. Compared with the Ctrl group, CPE supplementation increased BW (*p* < 0.05) and ADG (*p* < 0.01) on d14, but decreased F/G on d14 (*p* < 0.01).

### 3.3. Dietary CPE Supplementation Improved Intestinal Morphology of Early Weaning Piglets

**Figure 1:**
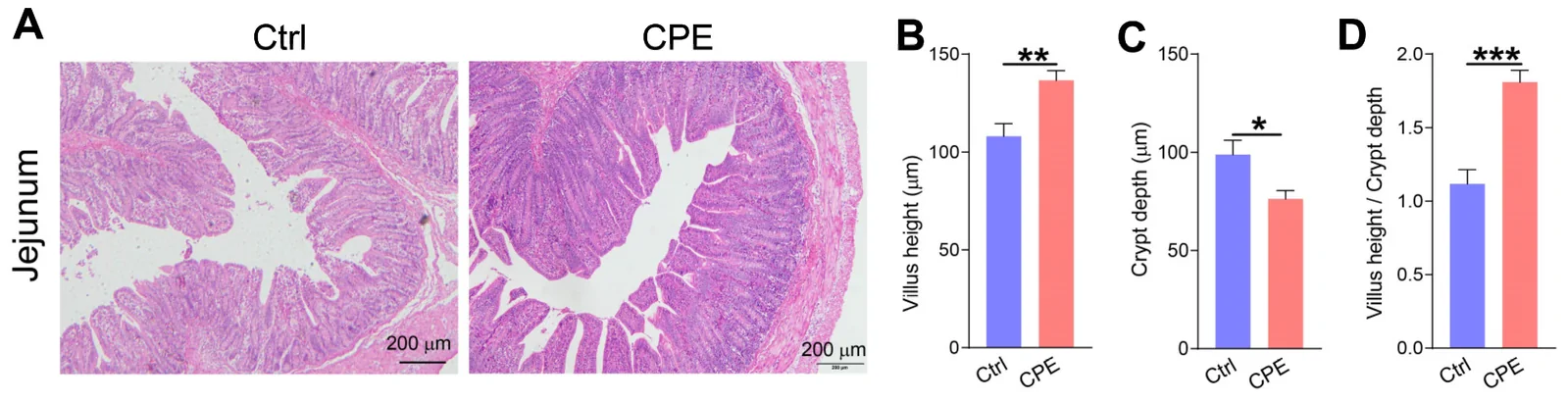
The effect of CPE supplementation on jejunum morphology in weaning piglets. (**A**) representative H&E-stained jejunum sections, (**B**) villus height, (**C**) crypt depth, and (**D**) villus height/crypt depth ratio of the jejunum. Data presented as mean ± SD. Ctrl group, piglets were fed the basal diet; CPE, Phellodendri extract; CPE group, piglets were fed the basal diet supplemented with 0.04% CPE. Asterisks indicate significant differences: * *p* < 0.05, ** *p* < 0.01 and *** *p* < 0.001.

The intestinal morphology in early weaning piglets is presented in Figure 1. Compared with the Ctrl group, CPE supplementation improved the jejunum morphology, (Figure 1A) indicated by the increased villus height (*p* < 0.01) (Figure 1B), decreased crypt depth (*p* < 0.05) (Figure 1C), and increased villus height/crypt depth ratio (*p* < 0.001) (Figure 1D).

### 3.4. Dietary CPE Supplementation Improved Intestinal Redox Imbalance of Early Weaning Piglets

**Figure 2:**
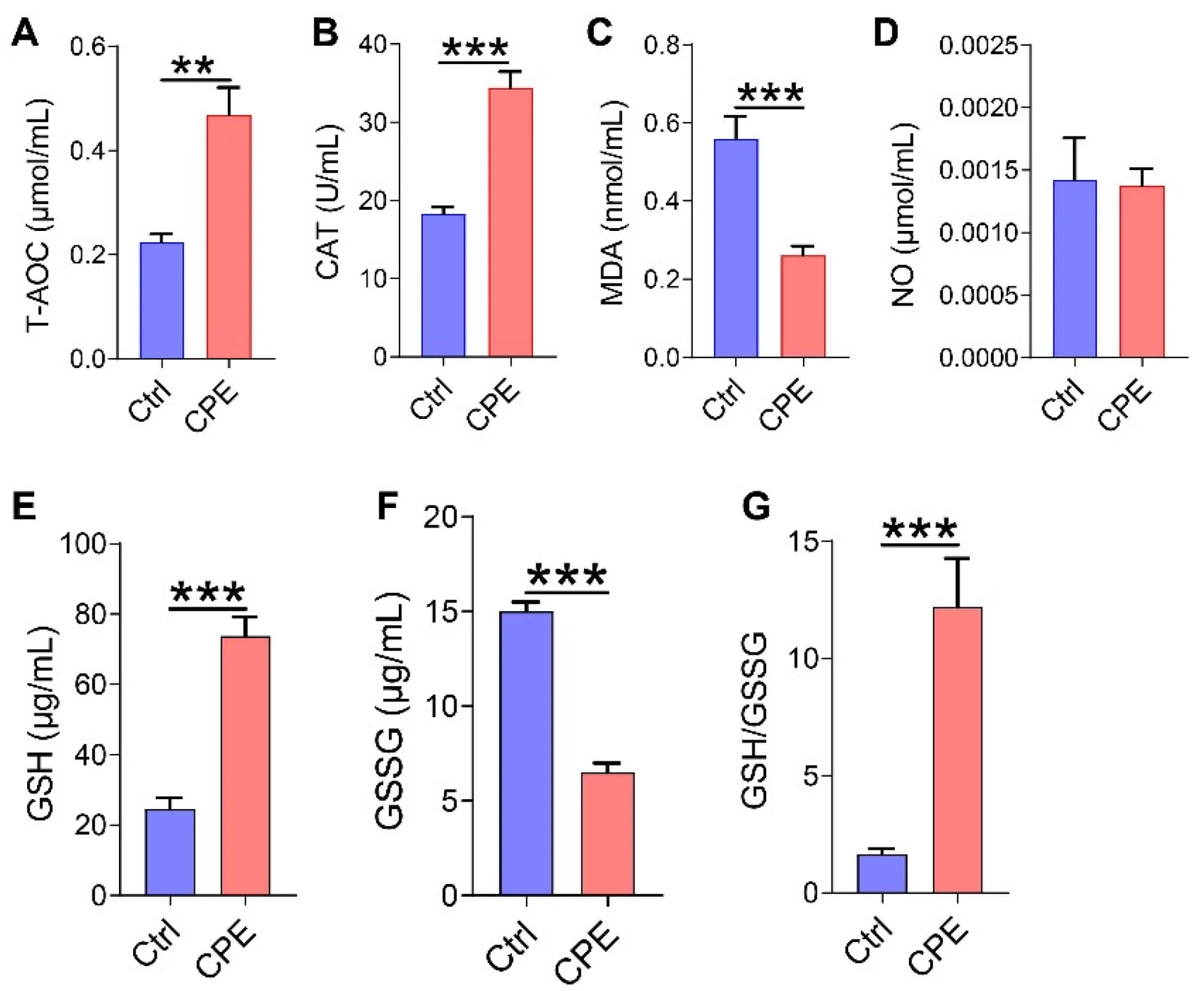
The effect of CPE supplementation on the oxidative stress indices in the piglets’ jejunum tissue. The oxidative stress indices include (**A**) T-AOC, (**B**) CAT, (**C**) MDA, (**D**) NO, (**E**) GSH, (**F**) GSSG, and (**G**) GSH/GSSG in the piglets’ jejunum tissue. Data presented as mean ± SD. Ctrl group, piglets were fed the basal diet; CPE, *Phellodendri extract*; T-AOC, total antioxidant capacity; GSH, glutathione; GSSG, glutathione disulfide; MDA, malondialdehyde; CAT, catalase; NO, nitric oxide. CPE group, piglets were fed the basal diet supplemented with 0.04% CPE. Asterisks indicate significant differences: ** *p* < 0.01 and *** *p* < 0.001.

The effects of dietary CPE supplementation on early weaning piglets’ intestinal redox imbalance are shown in Figure 2. Compared with the Ctrl group, CPE increased antioxidant indices including GSH (Figure 2E, *p* < 0.001), GSH/GSSG (Figure 2D, *p* < 0.001), T-AOC (Figure 2A, *p* < 0.01), and CAT (Figure 2B, *p* < 0.001), whereas it reduced oxidative metabolite MDA (Figure 2C, *p* < 0.001) and GSSG (Figure 2G, *p* < 0.001) in jejunum tissue. CPE supplementation had no significant effect on NO (Figure 2D, *p* > 0.05). These results indicated that dietary CPE supplementation improved intestinal barrier redox imbalance of early weaning piglets.

### 3.5. Dietary CPE Supplementation Regulated Early Weaning Piglets’ Intestinal Gene Expression of Inflammatory Cytokines and Antioxidant Indices

**Figure 3:**
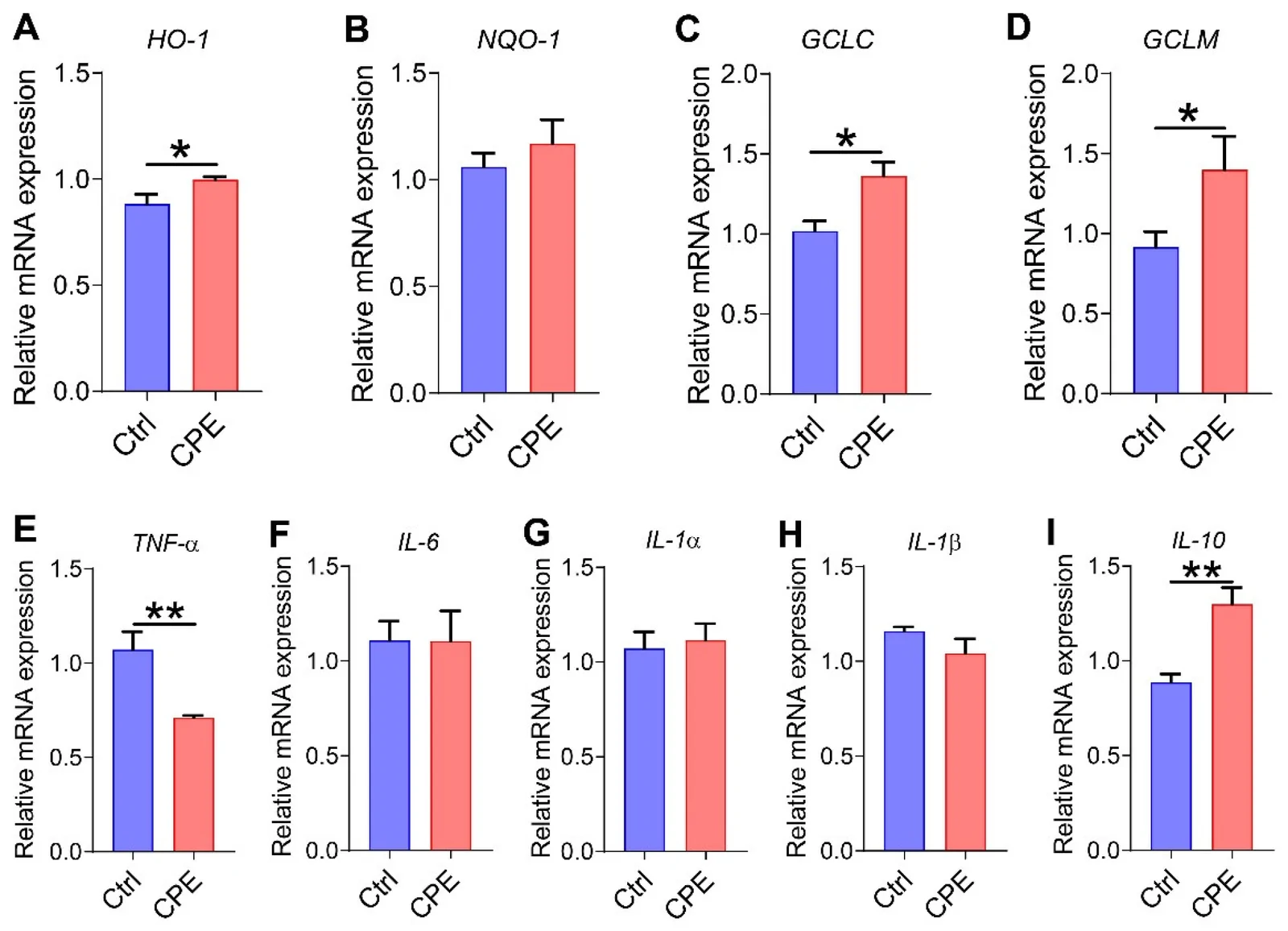
The effect of CPE supplementation on the gene expression levels of inflammatory cytokines and antioxidant factors in early weaning piglets. The antioxidant factors genes including (**A**) HO-1, (**B**) NQO-1, (**C**) GCLC, and (**D**) GCLM; the inflammatory cytokines genes including (**E**) TNF-α, (**F**) IL-6, (**G**) IL-1α, (**H**) IL-1β, and (**I**) IL-10. Data presented as mean ± SD. Ctrl group, piglets were fed the basal diet; CPE, *Phellodendri extract*; CPE group, piglets were fed the basal diet supplemented with 0.04% CPE. Asterisks indicate significant differences: * *p* < 0.05 and ** *p* < 0.01. HO-1, heme oxygenase-1; NQO-1, NAD(P)H quinone oxidoreductase 1; GCLC, glutamate–cysteine ligase catalytic subunit; GCLM, glutamate–cysteine ligase modifier subunit.

The genes expression levels of antioxidant indices and inflammatory factors in the jejunum tissue are shown in Figure 3. The results indicated that CPE improved early weaning piglets’ antioxidant capacity by increasing the mRNA levels of HO-1 (Figure 3A, *p* < 0.05), GCLC (Figure 3C, *p* < 0.05), and GCLM (Figure 3D, *p* < 0.05) in jejunum tissues compared with the Ctrl group. CPE increased the mRNA levels of the anti-inflammatory cytokine IL-10 (Figure 3I, *p* < 0.05) but decreased inflammatory cytokine TNF-α (Figure 3E, *p* < 0.01) compared with the Ctrl group. Overall, CPE enhanced the antioxidative capacity and improved the inflammation of jejunum tissues in early weaning piglets.

### 3.6. Dietary CPE Supplementation Shifted Early Weaning Piglets’ Gut Microbial Structure and Diversity

**Figure 4:**
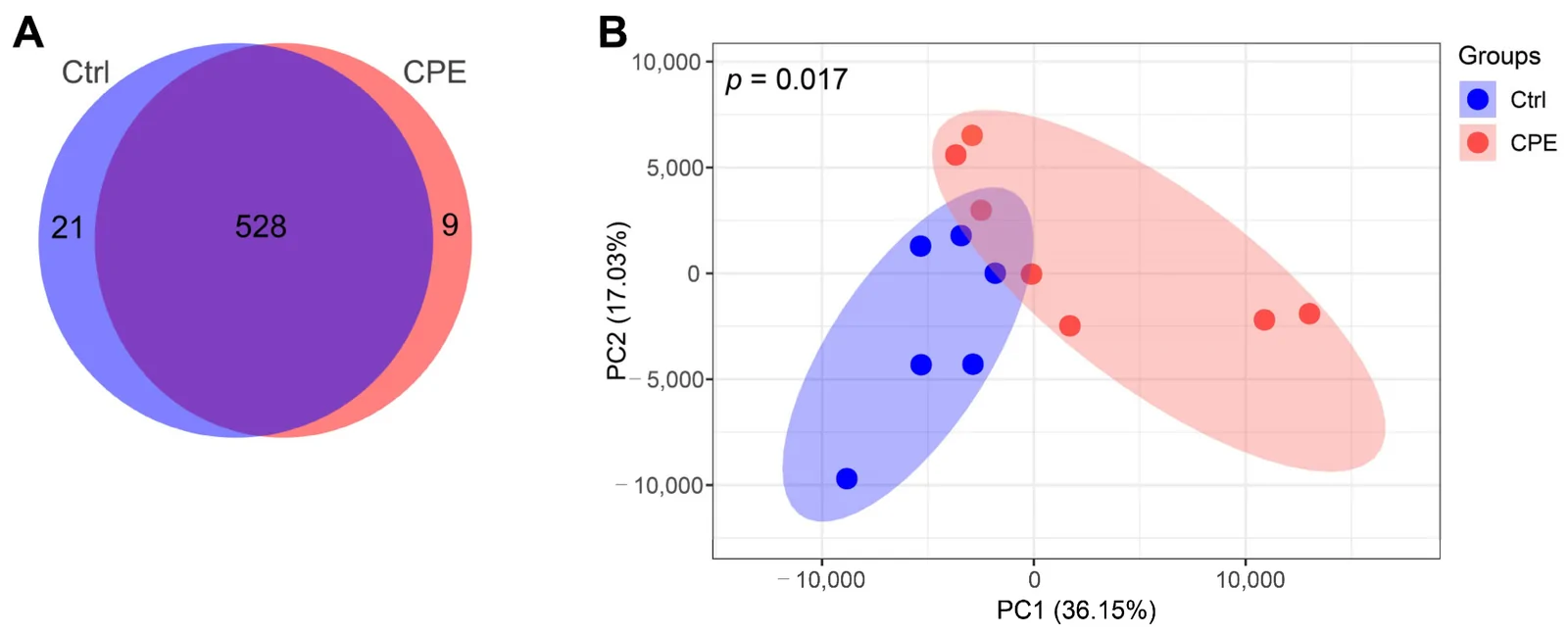
The effect of CPE supplementation on the diversity of gut microbiota in early weaning piglets. (**A**) Venn diagram of OTU, (**B**) principal co-ordinate (PCoA) plot based on UniFrac distance matrix between groups. Ctrl group, piglets were fed the basal diet; CPE, *Phellodendri extract*; CPE group, piglets were fed the basal diet supplemented with 0.04% CPE.

As shown in Figure 4, the OTU number distribution among the groups showed that CPE had a different OTU catalog compared with the Ctrl group (Figure 4A), indicating that the gut microbial structures of Ctrl and CPE were different. PCoA plots assessed by PERMANOVA analysis showed that the gut microbiota among groups were significantly different (Figure 4B, *p* = 0.017). Alpha diversity indices indicating the diversity of gut microbiota in a single sample showed that CPE increased alpha diversity indices— Observed species (*p* < 0.05) and ACE (*p* < 0.05), but had no effect on Shannon, Simpson, and Coverage than the Ctrl group (Table 3).

### 3.7. Dietary CPE Supplementation Shifted the Relative Abundance of Early Weaning Piglets’ Gut Microbiota

**Figure 5:**
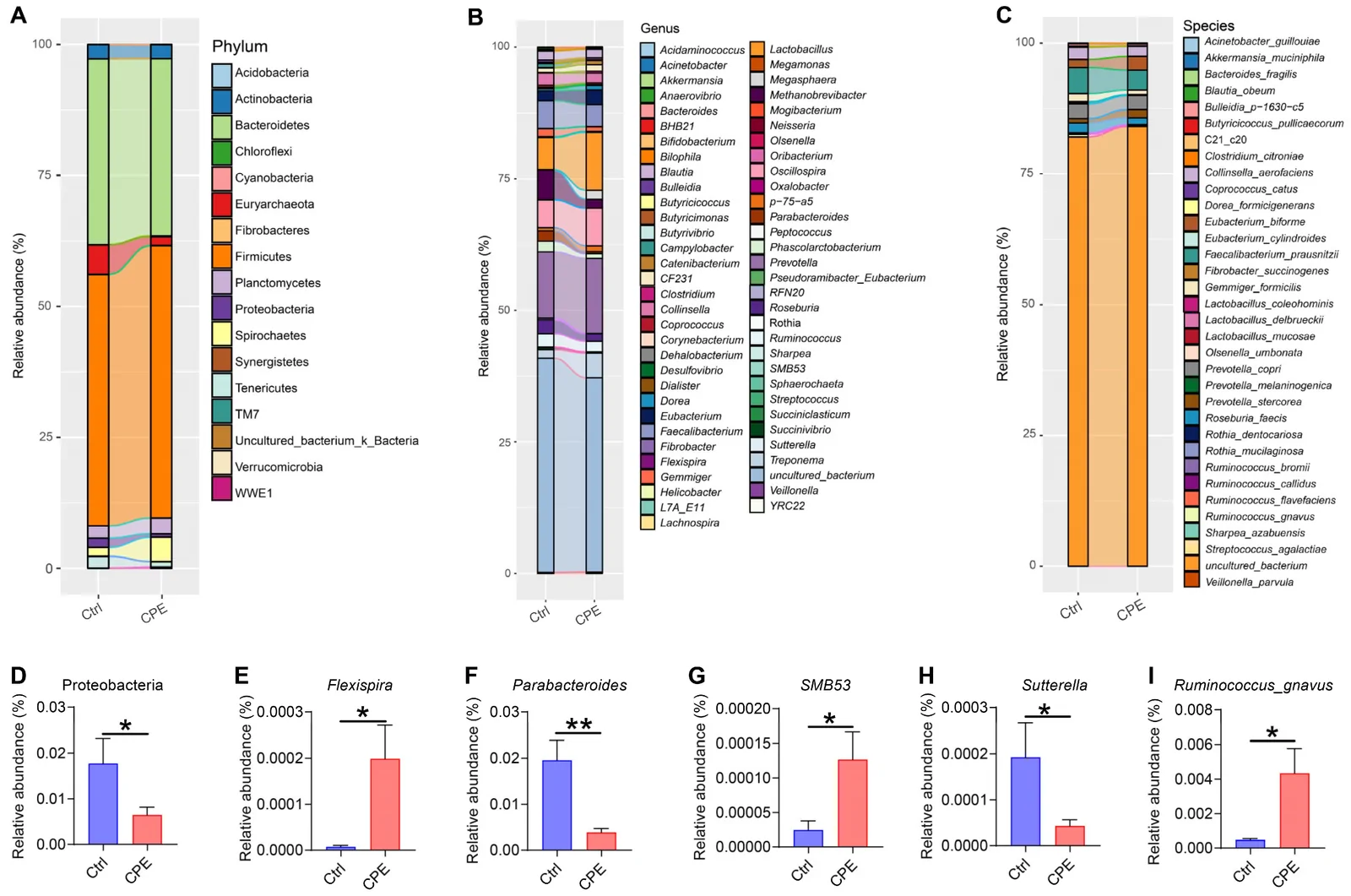
The effect of CPE supplementation on the composition of gut microbiota in early weaning piglets. The relative abundances of (**A**) phylum, (**B**) genus, and (**C**) species. (**D**–**I**) The bacteria differed significantly among the groups. Data presented as mean ± SD. Ctrl group, piglets were fed the basal diet; CPE, *Phellodendri extract*; CPE group, piglets were fed the basal diet supplemented with 0.04% CPE. Asterisks indicate significant differences: * *p* < 0.05 and ** *p* < 0.01.

The linked bar plots of the taxon abundance illustrated that dietary CPE supplementation markedly shifted the relative abundance of bacteria at different taxon levels including phylum (Figure 5A), genus (Figure 5B), and species (Figure 5C). Compared with the Ctrl group, CPE increased the relative abundances of Proteobacteria (*p* < 0.05, Figure 5D), *SMB53* (*p* < 0.05, Figure 5G), *Flexispira* (*p* < 0.05, Figure 5E), and *Ruminococcus_gnavus* (*p* < 0.05, Figure 5I), but decreased the relative abundances of Parabacteroides (*p* < 0.01, Figure 5F) and *Sutterella* (*p* < 0.05, Figure 5H).

### 3.8. Gut Microbiota Shifted by Dietary CPE Supplementation Has a Significant Correlation with the Early Weaning-Related Indices

**Figure 6:**
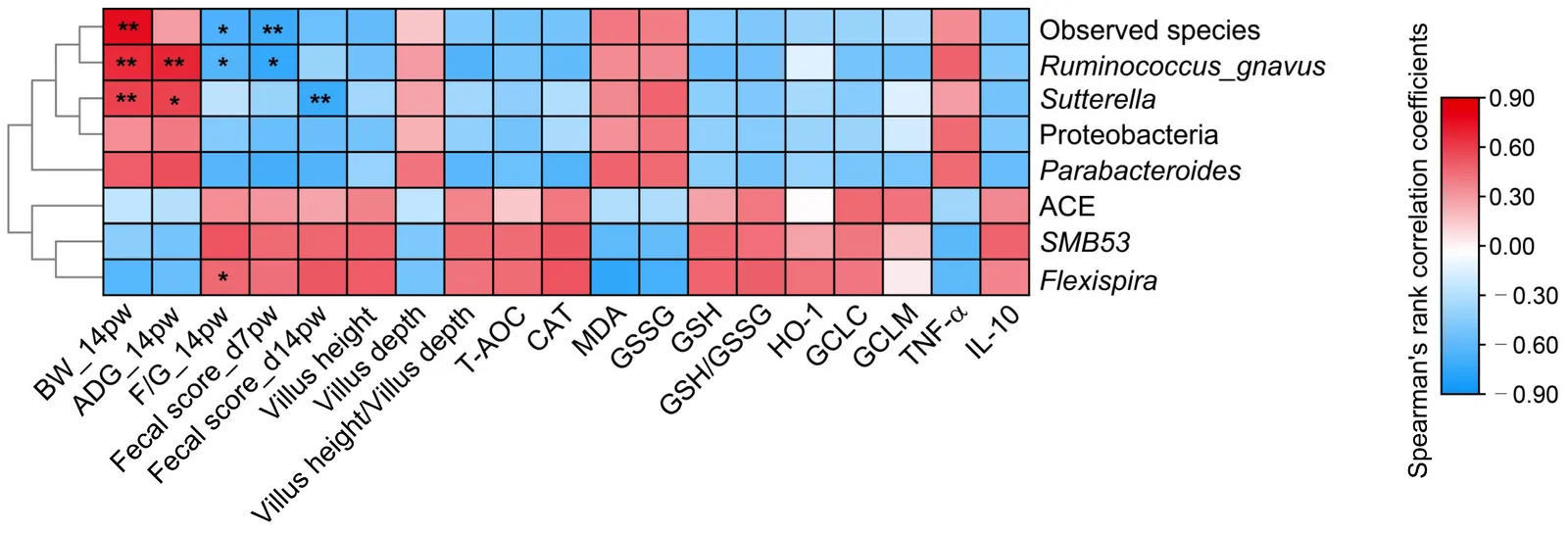
Heat map of the Spearman rank correlation coefficient between the CPE-responded bacteria and the early weaning indices. The blue color represents a negative correlation while red represents a positive correlation. Asterisks indicate significant differences: * *p* < 0.05 and ** *p* < 0.01. ADG, average daily weight gain; BW, body weight; CAT, catalase; F/G ratio, ratio of feed to gain; GCLC, glutamate–cysteine ligase catalytic subunit; GCLM, glutamate–cysteine ligase modifier subunit; GSH, glutathione; GSSG, glutathione disulfide; HO-1, heme oxygenase-1; MDA, malondialdehyde; T-AOC, total antioxidant capacity; 7pw, day 7 post-weaning; 14pw, day 14 post-weaning.

The Spearman rank correlation coefficient and significance testing (Figure 6) showed that alpha diversity index, Observed species, was negatively correlated with F/G_14pw (*p* < 0.05) and Fecal score_d7pw (*p* < 0.01); but was positively correlated with BW_14pw (*p* < 0.01). *Ruminococcus_gnavus* was positively correlated with BW_14pw (*p* < 0.01) and ADG_14pw (*p* < 0.01), but was negatively correlated with F/G_14pw (*p* < 0.05) and Fecal score_d7pw (*p* < 0.05). *Sutterella* was positively correlated with BW_14pw (*p* < 0.01) and ADG_14pw (*p* < 0.05), but was negatively correlated with Fecal score_d14pw (*p* < 0.01). Flexispira was positively correlated with F/G_14pw (*p* < 0.05).

## 4. Discussion

Through dietary supplementation with CPE, we found that dietary 0.04% CPE and 0.08% CPE supplementation gained a comparable effect on attenuating early weaning piglets’ diarrhea on d7 and d14 post-weaning. This is consistent with our prior experiment’s conclusion that CPE can effectively ameliorate post-weaning piglets’ diarrhea [22]. Piglets’ early weaning accompanied by shifts in diet, social relationship, and environment is frequently associated with transient anorexia, growth performance impairment, severe intestinal damage, infections, and gut microbiota dysbiosis [3,4]. Therefore, we determined the effect of dietary 0.04% CPE supplementation using the above indices and found that CPE supplementation significantly improved growth performance only during days 8–14, rather than immediately in the first week post-weaning, is consistent with the known pathophysiology of early weaning stress. During the first week of post-weaning, piglets undergo a period of transient anorexia and intestinal adaptation, during which the acute effects of stress (e.g., reduced feed intake, villus atrophy, and microbial dysbiosis) are most severe [2,3,4]. The beneficial effects of CPE on intestinal morphology, oxidative stress, and inflammation likely require several days to manifest and to translate into measurable improvements in growth performance. Thus, the significant improvement observed during the second week post-weaning likely reflects the cumulative effects of CPE on gut health restoration and nutrient absorption.

Early weaning stress usually results in small intestine atrophy, intestinal oxidative stress, and intestinal inflammation [2]. Here, our data showed that dietary CPE supplementation improved intestinal morphology of early weaning piglets by increasing villus height and villus height/crypt depth ratio. Zhu et al. and Du et al. reported that berberine of CPE has a protective effect on alleviating ETEC-induced gut injury in piglets [31,32]. As for intestinal oxidative stress, dietary CPE supplementation improved intestinal redox imbalance of early weaning piglets by increasing antioxidant indices including GSH, GSH/GSSG, T-AOC, and CAT, whereas reducing oxidative metabolite MDA and GSSG in jejunum tissue. Meanwhile, dietary CPE supplementation improved early weaning piglets’ antioxidant capacity by increasing the mRNA levels of HO-1, GCLC, and GCLM in jejunum tissues. In addition, dietary CPE supplementation increased the mRNA levels of the anti-inflammatory cytokine IL-10 but decreased inflammatory cytokine TNF-α. The Nrf2 signaling pathway serves as a central defender against oxidative injury, not only by limiting the detrimental effects of reactive oxygen species but also by preserving intestinal barrier homeostasis through coordinated regulation of pro-inflammatory mediators and antioxidant enzymes [33]. Upon oxidative challenge, Nrf2 translocates to the nucleus and activates a panel of cytoprotective genes, including the well-characterized targets HO-1 and NQO1 [34]. In the present study, we observed that supplementing the diet with CPE significantly elevated the jejunal mRNA levels of HO-1, GCLC, and GCLM—the latter two encoding the catalytic and modulatory subunits of glutamate–cysteine ligase (GCL), which catalyzes the rate-limiting step in glutathione (GSH) biosynthesis [35,36]. These transcriptional changes were consistent with the improved antioxidant status measured in the jejunum. In agreement with our observations, berberine—the principal bioactive component of CPE—has also been reported to attenuate ETEC-induced intestinal inflammation and oxidative injury in weaned piglets, an effect linked to its modulatory action on the gut microbiota [37]. Overall, dietary CPE supplementation enhanced the antioxidative capacity and improved the inflammation of jejunum tissues in early weaning piglets.

Early weaning stress can result in piglets’ gut microbiota dysbiosis which could be involved in the etiology of piglets’ diarrhea and enteric dysfunction [2,38]. Inflammatory conditions within the intestinal lumen create a state of oxidative stress that can disrupt the local oxygen environment, thereby altering the ecological niches available to the commensal microbiota and promoting microbial imbalance [2,39]. To investigate whether CPE influences this process, we performed 16S rDNA sequencing on colonic samples from piglets. Our results revealed that dietary CPE not only modified the overall composition of the gut microbial community but also enhanced species richness, as reflected by increased Observed species and ACE indices. This gut microbiota-shift effect of CPE also had been found in broilers infected with *Salmonella enteritidis* [40]. Comparatively, berberine, the main active constituent of CPE also optimizes intestinal microbial composition in a weaned piglet model [32,37]. Further taxon analysis showed that dietary CPE supplementation increased the relative abundances of Proteobacteria, *SMB53*, *Flexispira*, and *Ruminococcus_gnavus*, but decreased the relative abundances of *Parabacteroides* and *Sutterella*. Certain bacterial taxa have been functionally linked to host metabolism and behavior; for instance, colonization by Proteobacteria can influence both metabolic pathways and neurobehavioral responses to cocaine [41]. Meanwhile, the genus *SMB53* has been positively correlated with body weight gain [42]. *Flexispira*, a pathogen closely related to Helicobacter, has been implicated in persistent bacteremia among individuals with primary immunodeficiency disorders [43]. *Ruminococcus gnavus* exhibits a dual-role association with various intestinal and extraintestinal conditions, ranging from inflammatory bowel diseases to neurological disorders [44]. *Parabacteroides* is noted for its ability to selectively utilize medicinal polysaccharides [45], while *Sutterella* and its associated metabolic pathways have been positively linked to vaccine-induced antibody responses in infant rhesus macaques [46]. Given that the sudden change in the form and composition of the diet of weaned piglets from liquid breast milk to solid feed can harm intestinal function and gut microbiota [47], we conducted Spearman’s rank correlation analysis and further revealed that these distinct microbial taxa were significantly correlated with the measured parameters, suggesting that the gut microbiota contributes to the beneficial effects of CPE in alleviating oxidative stress, inflammation, and microbial dysbiosis during early weaning in piglets. Therefore, supplementing CPE may be a potential strategy to facilitate early weaning transition in piglets by alleviating early weaning-related syndromes such as swine inflammation and necrosis syndrome [47].

## 5. Conclusions

This study represents the first comprehensive analysis of the CPE on facilitating piglets’ early weaning transition and found that dietary 0.04% CPE supplementation could effectively improve piglets’ fecal scores and growth performance. In terms of mechanism, CPE supplementation alleviated intestinal oxidative stress, inflammation, and microbiota dysbiosis in early weaning piglets. Further studies clarifying the role and mechanism of CPE-shifted gut microbiota in facilitating piglets’ early weaning transition will be essential.

## Figures and Tables

**Table 1:** Effects of CPE on the fecal scores of early weaning piglets ^1^.

Item	Ctrl	0.04% CPE	0.08% CPE
Fecal scores			
d7 post-weaning	3.00 ± 0.65 ^a^	2.13 ± 0.73 ^b^	2.00 ± 0.71 ^b^
d14 post-weaning	2.50 ± 0.58 ^a^	1.63 ± 0.45 ^b^	1.90 ± 0.48 ^b^

^1^ d7, 7 days post-weaning; d14, 14 days post-weaning; Ctrl group, piglets were fed the basal diet; CPE, *Phellodendri extract*; 0.04% CPE group, piglets were fed the basal diet supplemented with 0.04% CPE; 0.08% CPE group, piglets were fed the basal diet supplemented with 0.08% CPE. ^ab^ Within a row, values with a different superscript letter are significantly different (*p* < 0.05).

**Table 2:** Effects of CPE supplementation on the growth performance of early weaning piglets ^1^.

Item	Ctrl	CPE	*p*-Value
Day 0 to 7			
BW at d0, kg	6.35 ± 0.46	6.47 ± 0.56	0.69
BW at d7, kg	6.94 ± 0.45	7.11 ± 0.48	0.54
ADFI, g	145.59 ± 24.91	150.58 ± 30.34	0.75
ADG, g	84.50 ± 18.20	91.37 ± 25.27	0.57
F/G ratio	2.13 ± 0.06	1.74 ± 0.51	0.07
Day 8 to 14			
BW at d14, kg	7.67± 0.50	8.21 ± 0.30	0.040
ADFI, g	171.94 ± 17.06	186.50 ± 30.68	0.36
ADG, g	103.31 ± 22.70	157.63 ± 32.95	0.0037
F/G ratio	1.74 ± 0.28	1.21 ± 0.19	0.0024

^1^ ADFI, average daily feed intake; ADG, average daily weight gain; BW, body weight; F/G ratio, ratio of feed to gain; d 0, the day of weaning; d7, day 7 post-weaning; d14, day 14 post-weaning; CPE, *Phellodendri extract*. Data presented as mean ± SD. Ctrl group, piglets were fed the basal diet; CPE group, piglets were fed the basal diet supplemented with 0.04% CPE.

**Table 3:** Effects of CPE on the gut microbial diversity of weaning piglets ^1^.

Item	Ctrl	CPE	*p*-Value
Alpha_diversity			
Observed species	397.83 ± 29.45	442.29 ± 22.44	0.016
ACE	440.82 ± 24.98	478.60 ± 19.59	0.017
Chao1	451.41 ± 31.19	488.38 ± 28.97	0.067
Simpson	0.96 ± 0.016	0.95 ± 0.020	0.74
Shannon	5.83 ± 0.50	5.79 ± 0.53	0.90
Coverage	0.999 ± 0.0001	0.99 ± 0.0002	0.98

^1^ Data presented as mean ± SD. Ctrl group, piglets were fed the basal diet; CPE, *Phellodendri extract*; CPE group, piglets were fed the basal diet supplemented with 0.04% CPE.

## Data Availability

The original data presented in this study are included in the article. Further inquiries are available from the corresponding author upon reasonable request.

## Supplementary Materials

The following supporting information can be downloaded at: https://www.mdpi.com/article/10.3390/ani16162628/s1, Table S1. Ingredients and nutrients levels of basal diets (as-fed basis, %). Table S2. Primers sequences of target genes selected for analysis by real-time.

## Abbreviations

The following abbreviations are used in this manuscript:

ADG	Average daily gain
ADFI	Average daily feed intake
T-AOC	Total antioxidant capacity
CAT	Catalase
MDA	Malondialdehyde
GSH	Glutathione
NO	Nitric oxide
GSSG	Glutathione disulfide
BW	Body weight
