# Supplementary material for: Cortex Phellodendri Extract Supplementation Alleviates Intestinal Oxidative Stress, Inflammation, and Microbiota Dysbiosis in Early Weaning Piglets
Source: Animals (Basel). 2026 Aug 21;16(16):2628. doi: 10.3390/ani16162628 (PMC13509673; doi:10.3390/ani16162628)
Supplement: Supplementary file 1 [file animals-16-02628-s001.zip › animals-4490241-supplementary.pdf]

Table S1. Ingredients and nutrients levels of basal diets (as-fed basis, %).

Item	Content
Ingredients	
Corn	20.02
Soybean meal	8.00
Expanded soy bran	10.00
Expanded corn	35.00
Fermented soybean meal	7.00
Soybean oil	1.00
Sucrose	3.00
50% Intestinal membrane protein powder	3.00
Low protein whey powder	6.00
Fish meal	3.00
L-Lysine HCl	0.45
DL-Methionine	0.15
L-Threonine	0.15
L-Tryptophan	0.03
Choline chloride	0.10
Limestone	0.90
Dicalcium phosphate	0.90
NaCl	0.30
Vitamin and mineral premix[1]	1.00
Nutrient levels	
DM	88.57
DE, MJ/kg	14.35
CP	19.01
Ca	0.81
Total P	0.58
Available P	0.42
Lysine	1.41
Methionine	0.46
Methionine + Cystine	0.73
Threonine	0.87
Tryptophan	0.25

[1] Provided per kilogram of complete diet; vitamin A: 1,5500 IU; vitamin D_3: 3,000 IU; vitamin E: 40.0 mg; vitamin K: 1 mg; vitamin B_1: 4.5 mg; vitamin B_2:10.5 mg; vitamin B_6: 7 mg; vitamin B_{12}: 0.04 mg; folic acid: 2.0 mg; nicotinamide: 45 mg; D-biotin: 0.3 mg; D-pantothenic acid: 25 mg; Fe (as $FeSO_4 \bullet H_2O$), 100 mg; Cu (as $CuSO_4 \bullet 5H_2O$), 6 mg; Mn (as $MnSO_4 \bullet 5H_2O$), 20 mg; Zn (as $ZnSO_4 \bullet 7H_2O$), 90 mg; I (as KI), 0.14 mg; Se (as $Na_2SeO3 \bullet 5H_2O$), 0.30 mg.

Table S2. Primers sequences of target genes selected for analysis by real-time.

Gene	Primer sequences (5′-3′)	Size, bp	Temperature, °C
TNF-a	F: ACCTCCTCTCTGCCATCAAG	173	60
	R: CTGCCCAGATTCAGCAAAGT		
IL-1b	F: ATTCAGGGACCCTACCCTCTC	92	60
	R: ATCACTTCCTTGGCGGGTTC		
IL-1a	F: GCATGTGCTGAGCCTTTGTA	181	60
	R: CCTGGTCCTCCCAAGATTGT		
IL-6	F: GATGCTTCCAATCTGGGTTCA	62	58
	R: CACAAGACCGGTGGTGATTC		
IL-10	F: CGGCCCAGTGAAGAGTTTCT	98	60

	R: GGCAACCCAGGTAACCCTTA		
SOD	F: GAGACCTGGGCAATGTGACT	118	60
	R: CTGCCCAAGTCATCTGGTTT		
GCLC	F: CAAACCATCCTACCCTTTGG	172	58
	R: ATTGTGCAGAGAGCCTGGTT		
GCLM	F: GATGCCGCCCGATTTAACTG	159	58
	R: ACAATGACCGAGTACCGCAG		
HO-1	F: CGCTCCCGAATGAACACTCT	148	60
	R: GCGAGGGTCTCTGGTCCTTA		
NQO-1	F: ATCACAGGTAAACTGAAGGACCC	229	60
	R: TGGCAGCGTATGTGTAAGCA		
b-Actin	F: TCTGGCACCACACCTTCT	114	57
	R: TGATCTGGGTCATCTTCTCAC		

TNF-a = tumor necrosis factor-*a*; *IL* = interleukin; *SOD* = superoxide dismutase; *GCLC* = glutamate cysteine ligase catalytic subunit; *GCLM* = glutamate-cysteine ligase regulatory subunit; *HO-1* = heme oxygenase-1; *NQO-1* = phosphate adenine dinucleotide quinone oxidoreductase-1.
